# Ancient and Modern Hypnotism

**Published:** 1896-02

**Authors:** W. C. Achard

**Affiliations:** Gloucester, N. J.


THE
AMERICAN JOURNAL
—OF—
DENTAL SCIENCE.

VOL. XXIX. BALTIMORE, FEBRUARY, 1896. NO. 10.

ARTICLE I.

### ANCIENT AND MODERN HYPNOTISM.

BY. W. C. ACHARD, GLOUCESTER, N. J.

Read at the regular meeting of the Truman Dental Society, April 4, 1895.

I will endeavor to give a brief history of hypnotism, a description of the peculiar phenomena accompanying it, and a few words regarding its application in dentistry:

Hypnotism, of which the student of medicine has heard a great deal during the last few years, is by no means the new theory that many consider it; in fact, it is the very oldest method of curing diseases.

There is a sect in Egypt which has practiced it for over 4,000 years. Later on we have the Mumo-Jumbo-men of Africa, the medicimen men of the American Indian, the "charmers" of India, the Roman sibyls and Greek oracles. Of the last named we have, foremost, the oracle of Delphi, where in Apollo's temple a priestess sat for hours on a three-legged stool over a chasm in the ground, from which rose vapors of sulphur. In due time she entered the somnambulistic state, and prophesied to the awed listeners. Among the Israelites we have the well-known story of the "Witch of Endor," But of all followers of this most wonderful art, the fakirs in India surpass in skill all European hypnotizers. It is there where the greatest cures of diseases are effected, although accompanied with much unnecessary mummery.

In the middle ages there were the witches, which, in most cases, were poor unfortunates who had more than ordinary power of "animal magnetism," (as Mesmes called hypnotism). Then came, in 1662, Greatrakes, an Irishman, and a distinguished soldier, who had a revelation in a dream that he could cure scrofula by the laying on of hands. He accomplished some remarkable cures, and was followed, in 1773 by Gassner, an ex-monk of Suabia who traveled through southern Europe, using Latin pharses in hypnotizing his patients. But it remained for Mesmer to demonstrate the existence of "animal magnetism" in the human body.

Friedrich Anton Mesmer was born in 1734. After studying medicine in Vienna, he began his researches in "animal magnetism" the result of which I will give in his own words. He says: "Through certain manipulations, such as touching, stroking—in a word—magnetizing, even simply by merely a strong act of the will, one can produce this power in persons, impart it to others, and cause the most marvelous and wholesome effects." But he was considered a juggler by medical men, and expelled from Vienna, in 1774, for the alleged cure of a blind girl, (the case of Marie Paradise). He moved to Paris, and opened there his famous parlors. As it may interest you, I will describe his method for the treatment of all diseases: In the operating room there was, in the middle of the floor, a round tub, with a diameter of five feet, which was provided with a lid. On the bottom of it bottles were placed in such a way that the necks of some pointed to the center, and of others to the periphery. The tub was then filled with water to cover the bottles, and bent iron bars protruded through the lid. The walls were covered with mirrors, which, according to Mesmer's idea heightened the effect of the hypnosis. The patients were seated around the tub closely, and touching each other's knees, each holding one of the iron bars protruding through the lid, and also each other's hands. They were then connected by a cord, which was tied around their waists to assist in the passing of the "magnetic fluid." A mystic twilight pervaded the room, and the ear was charmed by sweet music of the harp and hiano, and also the soft melodies issuing from a harmonium, which Mesmer played with a master hand. After some time, the men and women sitting around the tub began to have peculiar sensations, as nervous twitchings, etc., and it was then that Mesmer entered solemnly, dressed in a fantastic robe of embroidered silk, holding in his hand an iron rod, with which he majestically walked around, stroking the patients. Some terrible crisis then ensued, the patients became hysterical, some having terrible convulsions. The most afflicted were then placed in a room, the floor and walls of which were padded.

It must be admitted that Mesmer really achieved some remarkable cures, which were discussed by the whole scientific world, and which led to the celebrated French Commission, appointed in 1784, by the government, to inquire into the nature of the new phenomena. This learned body of scientists included Franklin and Lavoisier, and the result of the investigation may be seen in the last words of this report: "Magnetism is one more fact in the history of human errors, and a great proof of the power of imagination." It may be stated, incidentally, that Mesmer closed his parlors to the commission, and they obtained their results from the parlors of one of his friends, Dr. Chas. D'Eslon. Mesmer then retired, and left France at the outbreak of the French Revolution, and died in England, in 1815.

Since then, hypnotism or mesmerism, as it was called has become an established fact. Prof. Charcot, the famous neurologist, of La Salpetriere, in Paris, commenced, in 1878, his investigations of the effect of hypnotism on hysterics, and demonstrated its different stages.

Another school is in Nancy, with Bernheim and Liebault at the head, and since July, 1886, there has been published a monthly journal, La Revue de L'Hypnotisme, with such contributors as Dr. E. Berillon, (the editor), Charcot, Luys, Voisin, Hack-Tuke, and others.

Hypnotism or mesmerism, of which somnambulism is the third stage, is that condition in which the will, self-consciousness, and sensations of the patient, are partially or entirely under the control of the operator, the subject losing, in most cases, all remembrance of what has passed during the hypnosis, but in which the subject, when hypnotized again, recovers the memory of the first hypnosis.

We will now consider the methods of producing hypnosis, which are as varied as the results. The ancient Egyptian sect, which I have mentioned, drew in the center of a white plate two triangles intersecting each other, and filled the figures with cabalistic words and signs. They then poured oil over the plate to make it shine more. After staring at the center of the plate for a few minutes, they entered the somnambulistic state. In Constantine, the members of the tribe of Beni-Aiaoussa sit down in a circle. After some time they begin peculiar swaying movements, until at last they spring up, their bodies dripping with perspiration, when with foaming mouths and in convulsions, they are absolutely insensible to pain, performing such feats as piercing their flesh with daggers, walking on hot irons, etc.

In Turkey, and Africa, there are to-day men who hypnotise themselves by staring intently upon their own umbilicus and pressing their tongue to the palate. After they are thoroughly under the hypnotic influence, they are put into an air-tight sack, which is sealed, then into a vault, which is also sealed and covered with earth, upon which grass is sowed. When the whole mound is covered with grass, the vault and sack are opened, and the man de-hypnotized, which is done by forcing open his mouth, pulling out his tongue, (which has settled into the pharynx), and then producing artificial respiration. Even after thus having been buried alive for months, the individual is said to be none the worse for his experience.

Other methods of producing hypnosis are to let the patient stare at a highly polished metal cap, or at a crystal, or any small object held at a distance of two or three centimeters before the eyes. A very good method, however, and one used by many operators, is Gessman's, which he describes as follows in his book, ' Magnetismus and Hypnotismus" : "I sit down opposite the patient, make her close her eyes, take her hands in mine, so that the four thumbs are pressed against each other, tell her to be quiet, and to yield unresistingly to the first inclination to sleep. When she has fallen asleep—generally within ten or twenty minutes—I increase the sleep by some strokings over her head and chest, and try to induce her to talk. This I easily achieve by placing one hand on her head and taking one of her hands in my other hand, while I, speaking towards the pit of her stomach, ask, 'Do you hear me?' which question must often be repeated four or five times before I receive a very weak answer." Now is the time for further experiments. Gessman uses a preliminary test to ascertain whether or not the patient is susceptible to hypnotism. This he does by telling her that in his organization there is a strong evolution of electricity, which enables him to electrize persons. He lets her seize with both hands two fingers on his right hand, and after some time asks if she feels anything. If she is susceptible, she feels a peculiar crawling sensation at first, and then a numbness in the upper part of the body. Then he says in commanding tone, "Hold my hand tight—tighter—tighter still—well! Now you cannot let go my hand;" and really she cannot. Blowing on the hands and telling her that she is free ends the spasm.

Thus could be enumerated many different methods; in fact, every operator has his own. Persons of a weak will are usually more susceptible than others. Take a child for example: When you, as a stranger, look at it fiercely and tell it "do this," it will do it, for it recognizes the superior will unconsciously, and that is only a mild form of hpy-notic suggestion.

The methods of de-hypnotizing vary also very much. You may use the same methods employed to revive a patient who has fainted. The simplest method is to blow on the forehead. Some persons are de-hypnotized by the simple command, "Awake!" Or, if you tell them that they shall count from one to ten, and awake when they say five, they will do so, and then keep on counting (not knowing why), or else let the word six die out on their lips.

The many phenomena of hypnotism have been divided into separate stages and classes by different authors. We will only consider Charcot's classification. He accepts the following three stages: First, the cataleptic; second, the lethargic, and third, the somnambulistic; which are described extensively by Dr. Paul Richter, a pupil of Charcot. The cataleptic state is produced by staring at some object, by a sudden strong light placed under the eyes, the sound of a gong, or a tremendous peal of thunder, also by sudden emotions, as fear, wrath, etc., when a person is for a shorter or longer time deprived of the power of speech or mobility. It also developes from the lethargic state when the already closed eyes are suddenly exposed to strong rays of light by the raising of the lids. It sometimes occurs before the narcotic state, during the administration of anæsthetics. A person who has been hypnotized is often very easily put in the cataleptic state by the clapping of the hands, the report of a gun, the sound of a gong, etc. An amusing incident illustrating this may be told here:

A woman had been suspected of stealing photographs from La Salpetriere, at Paris, but upon being questioned, she absolutely denied it. One day she was surprised, standing motionless at the place where the photographs were kept, with one hand stretched out grasping one. It seems that a gong was sounded in the next room to hypnotize a person there, and this woman almost immediately fell into the cataleptic state, which led to her discovery.

The characteristic of this state is immobility. The eyes are open and staring; respiration almost ceases; the limbs retain the most difficult positions in which they are placed; the skin is insensible to the strongest irritation, but the muscular senses, and those of hearing and vision partly retain their activity, and are susceptible to suggestion. Catalepsy ceases by a return to the normal condition, or by changing into the lethargic state. This state is produced by staring, or by continual pressure on the eyeballs. It can be produced from the cataleptic state by a simple closing of the eyelids. It is often preceded by epileptic phenomena, such as foaming mouth, asthmatic respiration of tetanus. The principle characteristics are complete insensibility of skin and mucous membrane, irritability of motor nerves and often insensibility to suggestion. Respiration is deep and quickened; limbs and body are perfectly relaxed. The muscles contract by stroking their tendon, and can also be excited directly. By opening the eyes the cataleptic state is reproduced.

The somnambulistic state is produced from the lethargic by a pressure or friction on the cranium. It is the one generally produced by magnetizers, and is characterized by absolute insensibility of the skin and mucous membrane. The senses are also sharpened, and the subject feels a certain personality, an ego, which may be illustrated by the following example: Before a patient in the cataleptic state are the requisits for shaving. They are touched by the operator and the patient at once begins to shave himself, but on being pressed on the cranium he stops and asks, "Why am I shaving myself?" I certainly do not need it!" On raising his eyelids he will go back to the cataleptic state and resume his shaving, but care must be taken that he does not cut himself, as he is absolutely insensible to pain. This state is produced by all methods which act on the imagination, and is especially susceptible to suggestion, which susceptibility is almost limitless, the actions of the somnambuld often bordering on the marvelous.

Among the physical effects of hypnotism we may name the muscular sensitiveness in different stages, varying from the flexibitis cerea in the cataleptic state, to the spasm and tetanus following it. Indeed, the rigidity of the muscles is at times so great that magnetizers produce the cruel experiment of suspending a person in that condition between two chairs, in such a way that the body rests only on the extremes of the head and feet; and to show this power over the subject, the magnetizer sits with his whole weight on the thus suspended body. This experiment is the more dangerous, as it may produce tetanus of the respiratory muscles. Analgesia, or entire insensibility to pain, seems to be a characteristic of all stages of hypnotism; with a few exceptions, in the somnambulistic state, where it is not only absent, but where the sensibility to pain is often very much increased. In this hyperæsthesia may be found an explanation why the somnambulist may recognize things with his eyes closed, or walk in the most dangerous places with absolute safety. The slightest touch with a hair is accurately localized by him.

In the cataleptic state, the sense of sight, hearing, smell, and taste, may be affected by suggestion, and very amusing experiments are made; as for example, one may give the person a green persimmon to eat, and tell her that it is a good apple, upon which she will enjoy it very much. Or on the other hand she may be given a good apple and told that she is eating a green persimmon, when she will immediately make a wry face and eject from the mouth what is stiil in it.

Among the mental effects, we have in the first place that on memory. It is a characteristic that the deeply hypnotized person upon awakening remembers nothing of what has passed during the hypnosis, which, however, can be controlled by the operator.

If, for instance, you tell your patient that you will remember everything that has passed upon awakening, he will do so. Very peculiar hallucination may be thus brought about. We will take an experiment made at Nancy, for illustration. Mrs. A. is hypnotised by staring. While asleep she is made to believe that three minutes after awaking she will go and embrace a little peasant woman who is sitting in the corner of the room, and whom she then sees for the first time. At the moment predicted she goes and embraces the woman, who is quite astonished at this unexpected caress. When a moment afterward she was asked, "What did you do?" she answered, "I? Nothing!" "Yes, you embraced that woman!" "No, certainly not!" She had already forgotten what she had done. From this it may be seen, that commands given during hypnosis will afterwards be carried out unconsciously, and this has led to many serious cases of criminal actions. We all remember the historic trial in Paris a few years ago, when a woman murdered a man in obedience to a command given her while under the hypnotic influence. And this brings to us the dangers and abuses of hypnotism.

I will only cite a few cases to show that the hypnotized can be injured by hypnotism, and that he can be used against or without his will as a tool for the service of crime. The sleep produced by hypnotism is not a normal one, but a nervous pathological state, and as all abnormal conditions of the body must injure it in time, it is obvious that rerepeated experiments on a sound and healthy patient are more or less injurious, and have a tendency to weaken not only the mind but the body. It may produce such a susceptibility that a subject will be brought immediately under the hypnotic influence by the slightest cause. There is a case on record in which a man became hypnotized on the street by a light from a carriage coming toward him, and he at once became cataleptic. Should that man walk on a railroad track and see the headlight of a locomotive, it may have the same effect on him, and he would lose his life.

The following is an experiment by Liegeois to prove that a person can be used for crime against his own will: A young man, 25 years old, while under hypnotic influence, was presented by Liegeois with a white powder, and told that it contained arsenic. "You will immediately go to your aunt; there you will take a glass of water, pour the arsenic into it, dissolve it well in water, and offer the poisoned drink to your aunt!" In the evening Liegeois was informed that the attempt at poisoning had been punctually performed. Thus we could enumerate many cases to show not only the good, but also the evils and abuses of this most wonderful art.

How can we apply it in medicine and dentistry? In medicine it has been used with great success all over the world. Insomnia, rheumatism, hysteria, etc., have been treated with its aid, and even amputations have been performed with absolute absence of pain. It may prove interesting to read Dr. James Esdaile's book on "Mesmerism in India," (London, 1846), in which, among other things, may be found a report of the medical and surgical cases treated by him with the aid of hypnotism.

In dentistry it has been used with more or less success. Many have been discouraged after the first few trials, but but those who have carefully and with untiring efforts sought the benefits obtained by its use, have been rewarded with greater success than they ever hoped for. We all have had patients whose fear and nervous excitement was so great that it was difficult even to make a proper examination of their teeth. Just there hypnotic suggestion may prove the greatest benefit. We can easily produce the first stage, and suggest quiet, absence of fear, and willingness to undergo any dental operation. This will aid the operator and patient very much, and hypnosis may be produced again after the operation, to suggest the same after the patient leaves the chair. An inexperienced operator may simply suggest a tranquil frame of mind for the time being and bad results may follow it. It is, therefore, essential to suggest that the patient shall feel absolute quiet and calm after the hypnosis, and shall not experience troublesome consequences. Too much stress cannot be laid on this; as the omission may bring on extreme nervousness, headache and disturbed sleep. The question now arises: Can hypnotic suggestion be used successfully to produce anaglesia during such minor dental operations as excavating cavities, extracting pulps, etc? Dr. Thomas Fillebrown, of Coston in his paper on "Hypnotic Suggestions as an Obtundent and Sedative," read at the World's Columbian Dental Congress, August 18, 1893, asserts that it can. For this purpose he indicates the first and second degree of the Nancy School, and by constant repetition of "Sleep, sleep, you are resting, you are not suffering, you are not dreading it, you do not care for it, sleep, sleep, etc., he maintains the hypnotic sleep, which otherwise might discontinue as soon as pain is produced. One especially good feature of employing hypnotism for obtunding sensitive dentine is, that the lightly hypnotised patient in most cases shrinks from pain, though he may not feel it, and thus we have a guide to avoid the pulp, which we have not in any other method of allaying pain.

Dr. Gomer Sandberg of Stockholm, issued in May, 1893, a pamphlet, Quelques, mots sur L'Hypnotisme, Stockholm, 1893, in which he describes the use of hypnotism for obtunding sensitive dentine in a case he had on the 20th of December, 1889. A lady came to him to have two teeth filled, but the extreme sensibility of the dentine did not permit even the touch of an excavator. He hypnotised the lady, and then suggested no agitation during the operation, quiet and insensibility of the teeth. The last words he repeated many times. He then commenced his work, which lasted one hour and a half, and during which she was perfectly quiet, not experiencing any pain whatever.

In conclusion, I will say that the practice of hypnotism requires a great deal of care and experience, and that the hypnotizer should have a good medical knowledge, which it may be said, the modern course in dentistry strives to impart to its student. Its study is not only interesting and instructive, but it is an art with which the good student of medicine and its various branches should make himself acquainted. With a firm will, and a determination to surmount all obstacles, the conscientious student of hypnotism, will pry into its mysteries, and, awed with wonder, exclaim with shakespeare:

"There are more things in heaven and earth
Than are dreamt of in your philosophy!"

—*Microcosm.*

